# Higher Brain Uptake of Gentamicin and Ceftazidime under Isoflurane Anesthesia Compared to Ketamine/Xylazine

**DOI:** 10.3390/pharmaceutics16010135

**Published:** 2024-01-19

**Authors:** Yeseul Ahn, Chanakya D. Patil, Ehsan Nozohouri, Sumaih Zoubi, Dhavalkumar Patel, Ulrich Bickel

**Affiliations:** 1 Department of Pharmaceutical Sciences, Jerry H. Hodge School of Pharmacy, Texas Tech University Health Sciences Center, Amarillo, TX 79106, USAszoubi@ttuhsc.edu (S.Z.); dhavalkumar.patel@ttuhsc.edu (D.P.); 2 Center for Blood-Brain Barrier Research, Jerry H. Hodge School of Pharmacy, Texas Tech University Health Sciences Center, Amarillo, TX 79106, USA

**Keywords:** ceftazidime, gentamicin, blood–brain barrier, permeability, isoflurane

## Abstract

We have recently shown that the volatile anesthetics isoflurane and sevoflurane acutely enhance the brain uptake of the hydrophilic markers sucrose and mannitol about two-fold from an awake condition, while the combined injection of the anesthetic agents ketamine and xylazine has no effect. The present study investigated two small-molecule hydrophilic drugs with potential neurotoxicity, the antibiotic agents ceftazidime and gentamicin. Transport studies using an in vitro blood–brain barrier (BBB) model, a monolayer of induced pluripotent stem cell-derived human brain microvascular endothelial cells seeded on Transwells, and LC-MS/MS analysis demonstrated the low permeability of both drugs in the range of sucrose, with permeability coefficients of 6.62 × 10^−7^ ± 2.34 × 10^−7^ cm/s for ceftazidime and 7.38 × 10^−7^ ± 2.29 × 10^−7^ cm/s for gentamicin. In vivo brain uptake studies of ceftazidime or gentamicin after IV doses of 25 mg/kg were performed in groups of 5–6 mice anesthetized at typical doses for surgical procedures with either isoflurane (1.5–2% *v*/*v*) or ketamine/xylazine (100:10 mg/kg I.P.). The brain uptake clearance, *K_in_*, for ceftazidime increased from 0.033 ± 0.003 μL min^−1^ g^−1^ in the ketamine/xylazine group to 0.057 ± 0.006 μL min^−1^ g^−1^ in the isoflurane group (*p* = 0.0001), and from 0.052 ± 0.016 μL min^−1^ g^−1^ to 0.101 ± 0.034 μL min^−1^ g^−1^ (*p* = 0.0005) for gentamicin. We did not test the dose dependency of the uptake, because neither ceftazidime nor gentamicin are known substrates of any active uptake or efflux transporters at the BBB. In conclusion, the present study extends our previous findings with permeability markers and suggests that inhalational anesthetic isoflurane increases the BBB permeability of hydrophilic small-molecule endobiotics or xenobiotics when compared to the injection of ketamine/xylazine. This may be of clinical relevance in the case of potential neurotoxic substances.

## 1. Introduction

**Figure 1:**
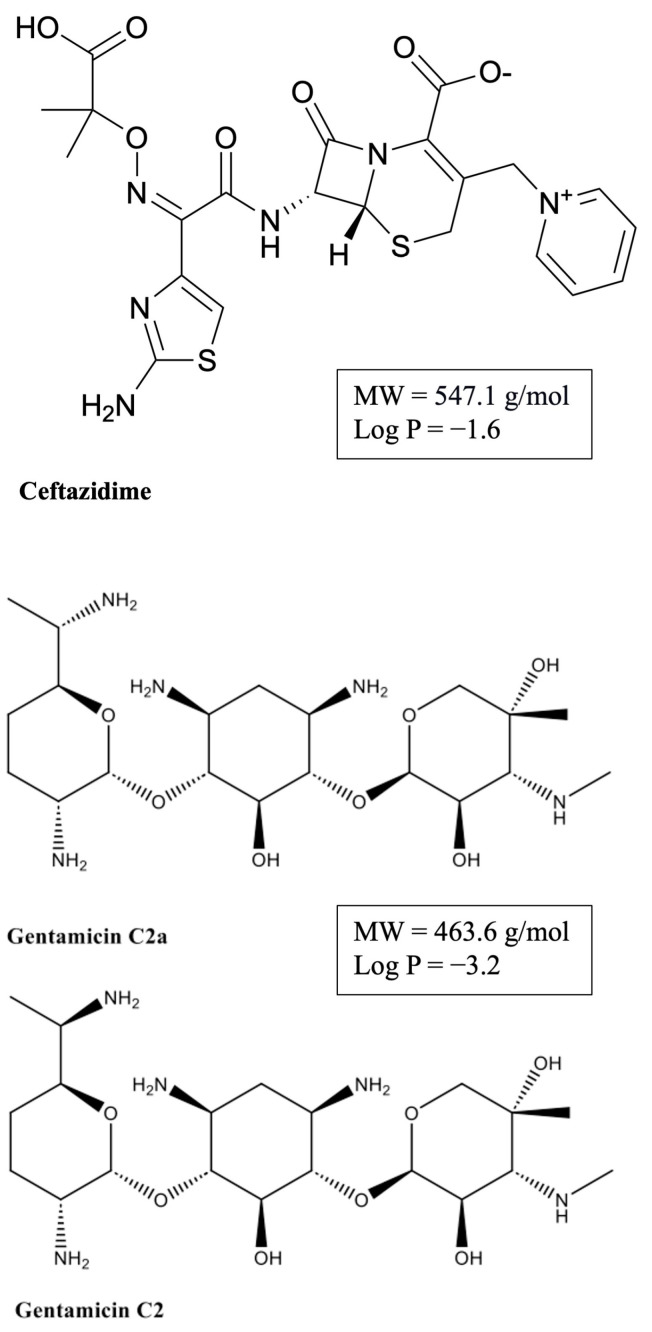
Structures of ceftazidime and the enantiomers gentamicin 2a and gentamicin 2.

The blood–brain barrier (BBB) maintains a stable microenvironment within the central nervous system (CNS) and is essential to protecting the brain against the entry of harmful endogenous substances and xenobiotics [1,2]. Some drugs of abuse (nicotine, cocaine, methamphetamine) are known to cause BBB dysfunction [3,4], and in recent years, there has been mounting evidence that volatile anesthetic agents in common clinical use, including isoflurane and sevoflurane, increase the permeability of the BBB [5,6,7,8,9,10]. While some of these studies investigated the influence of anesthesia on the BBB in the context of surgery [10] or traumatic brain injury [9], others studied the effects of the agents alone [5,6,7,8]. In order to characterize the principal mechanisms underlying alterations in BBB integrity by anesthetic agents, initially applying a reductionist in vitro approach with liposomes proved advantageous. Our group recently demonstrated increased fluidity in different lipid membranes at clinical concentrations of isoflurane (1 mM) using fluorescence anisotropy analysis [11]. In contrast, ketamine/xylazine at clinical concentrations does not affect lipid membranes [6]. Proceeding with an in vivo animal model in mice, we found that short exposure (30–45 min) to either isoflurane or sevoflurane, but not to ketamine/xylazine, caused an increase in BBB permeability. Both isoflurane and sevoflurane displayed a two-fold increase in the brain uptake clearance (apparent K_in_) of the hydrophilic marker [^13^C_12_]sucrose compared to either ketamine/xylazine anesthesia or an awake condition [6]. The expression of tight junction proteins (claudin-5, occludin and ZO-1) in the brain capillaries of these animals remained unchanged. Therefore, our data support the hypothesis that volatile anesthetic agents like isoflurane alter the lipid structure of cell membranes, transiently facilitating the brain uptake of otherwise poorly permeable, hydrophilic small molecules. Considering that these experiments were performed under clinically relevant concentrations of isoflurane, there is a possibility that potentially neurotoxic drugs, which are normally restricted by the BBB, may gain enhanced access to the CNS under inhalational anesthetics. In the present study, we assessed the permeability of the BBB to ceftazidime and gentamicin, two small hydrophilic drugs. Their molecular structures are depicted in Figure 1. Ceftazidime, a third-generation cephalosporin, is usually given together with a beta-lactamase inhibitor, avibactam, to treat complicated urinary tract infections and other infections caused by Gram-negative bacteria [12]. Cephalosporins are considered relatively safe drugs, but several cases of neurotoxicity have been reported in the literature. Most case reports are related to the later generations of cephalosporins, cefepime and ceftazidime, which are also strongly associated with renal impairment. The reported neurological manifestations caused by ceftazidime ranged from confusion and hallucinations to myoclonus and seizures [13,14,15]. The mechanism of cephalosporin-induced neurotoxicity has not been fully understood. It may involve γ-Aminobutyric acid type A(GABA-A) receptor inhibition [16], but to determine the electrophysiological effects, in vitro concentrations in the millimolar range were required [17]. Gentamicin, an aminoglycoside antibiotic, has been mostly associated with ototoxicity and nephrotoxicity [18]. Potential neurotoxicity involves N-methyl-D-aspartate (NMDA) receptor activation and may occur under pathophysiological conditions of increased BBB permeability [19,20]. Gentamicin is not a single compound, but is composed of a number of closely related molecules [21]. According to the USP, gentamicin C2 and C2a are major components, comprising 40% of the mixture. Gentamicin C2 and C2a are stereoisomers and have shown stable transition with reproducible results compared to gentamicin C1a [22]. Considering the above aspects, gentamicin C2 + C2a was chosen as the analyte of choice in the present study.

We first developed and validated highly sensitive and robust liquid chromatography–tandem mass spectrometry (LC-MS/MS) methods for the detection of ceftazidime and gentamicin in cell culture medium and in mouse plasma and brain. Subsequently, we used analytical methods to measure the BBB permeability of these small hydrophilic drugs in an in vitro BBB model using brain endothelial cells and in vivo in mice. For the in vivo study, mice were exposed to anesthesia with either isoflurane or ketamine/xylazine.

## 2. Materials and Methods

### 2.1. Chemicals and Reagents

Ceftazidime (Tazicef) for injection USP was obtained from Hospira Inc. (Lake Forest, IL, USA) and gentamicin injection USP was purchased from Fresenius Kabi (Lake Zurich, IL, USA).

Ceftazidime pentahydrate (>98% purity), formic acid (LC/MS grade, >98% purity), analytical-grade HFBA (heptafluorobutyric acid), TCA (trichloroacetic acid) and LC-MS/MS-grade acetonitrile and water were obtained from Fisher Scientific (Waltham, MA, USA). Meropenem trihydrate (>98% purity) was purchased from Alfa Aesar (Ward Hill, MA, USA). Tobramycin was obtained from Sigma Chemical (St. Louis, MO, USA), CD-1 mouse plasma from BioIVT Inc. (Westbury, NY, USA), heparin from APP Pharmaceuticals (Schaumburg, IL, USA), isoflurane from Piramal Critical Care (Bethlehem, PA, USA) and ketamine from Par Pharmaceutical Company Inc. (Spring Valley, NY, USA). All other chemicals were analytical grade and obtained from commercial and reliable sources.

### 2.2. Animals

Male C57Bl/6J mice aged 8–12 weeks and with a weight range of 22–32 g were purchased from Jackson Laboratories (Bar Harbor, ME, USA). The mice were housed in ventilated cages in a temperature- and humidity-controlled room with a 12 h light/12 h dark cycle and free access to standard rodent food and water. All animal procedures were approved and regulated in accordance with the Institutional Animal Care and Use Committee at Texas Tech University Health Sciences Center and complied with the National Research Council guidelines for the care and use of animals (National Research Council, 2011).

### 2.3. Mass Spectroscopic and Chromatographic Conditions

We modified published LC-MS techniques for ceftazidime [23] and gentamicin [24] as follows. An AB SCIEX QTRAP^®^ 5500 triple quadrupole mass spectrometer (MS) coupled with a Nexera UPLC system (Shimadzu, Kyoto, Japan) was used for mass spectrometric detection. The UPLC system accommodated an autosampler (Sil-30AC), pumps (LC-30AD), a controller (CBM-20A), a degasser (DGA-20A5) and a column oven (CTO-30A). Data acquisition and quantification were accomplished using Analyst software Version 1.7. Gradient separation chromatography for ceftazidime was carried out on a 2.6 µm Accucore C18 HPLC column (100 mm × 2.1 mm) (Thermo Fisher, Loughborough, UK; 17,126–102,130) with 0.1% formic acid in water (Mobile Phase A) and 0.1% formic acid in acetonitrile (Mobile phase B). A Kinetex 1.7 µm EVO C18 column (50 mm × 2.1 mm; Phenomenex, Torrance, CA, USA) was used for the chromatographic separation of gentamicin C2 + C2a. Analytical-grade HFBA (heptafluorobutyric acid) was used as an ion-pairing agent [25], for the retention of gentamicin on a C18 column. The gradient elution was performed using 20 mM HFBA in water (Mobile Phase A) and 20 mM HFBA in acetonitrile (Mobile phase B).

The optimized LC-MS/MS analytical method conditions for ceftazidime and gentamicin are summarized in Table 1.

### 2.4. Preparation of Standards and Quality Controls (QCs)

#### 2.4.1. Ceftazidime

Stock solutions of the antibiotic (1 mg/mL) were prepared by dissolving the powder in cell culture medium or diluting the injection solution in saline. Quality controls and standards were prepared by spiking plasma and diluted brain homogenate (1:9 *w*/*v* in LC-MS-grade water) with ceftazidime to make 100 µg/mL and 1000 ng/mL, respectively. The 100 µg/mL plasma sample was serially diluted with blank plasma, and each dilution was further diluted 100-fold with LC/MS-grade water to yield final concentrations of 10, 20, 50, 100, 250, 500 and 1000 ng/mL. For the brain standard samples, the 1000 ng/mL standard was further diluted using the diluted blank brain homogenate to obtain concentrations of 10, 20, 50, 100, 200, 400 and 1000 ng/mL. Amounts of 10, 100 and 1000 ng/mL were used for QCs in both plasma and brain. For the in vitro permeability study, the stock solution was processed by following the same protocol for the plasma standards and QCs, but cell culture medium (EC—for hBMEC was used instead of blank plasma to achieve concentrations of 10, 20, 50, 100, 250, 500 and 1000 ng/mL. All standards and QCs were then subjected to the sample preparation process described below.

#### 2.4.2. Gentamicin

For the plasma standard curve, 100-fold-diluted blank mouse plasma was spiked with gentamicin to produce a concentration of 1000 ng/mL, which was further diluted using 100-fold-diluted blank mouse plasma to achieve concentrations within the range of 10–1000 ng/mL. In the case of the brain standard curve, standard concentrations were prepared in LC-MS-grade water ranging from 100 to 10,000 ng/mL. These concentrations were then used to spike blank brain tissue homogenized in water (1:9 *w*/*v*) to produce a concentration range of 10–1000 ng/mL of gentamicin. Both plasma and brain standards were then processed using the sample preparation process described below.

### 2.5. LC-MS/MS Sample Preparation Process

#### 2.5.1. Ceftazidime

Plasma was diluted 100-fold in LC-MS/MS water, and brain was homogenized in LC-MS grade water (1:9 *w*/*v*). A total of 50 μL of diluted plasma or brain homogenate was added to 200 μL of water–acetonitrile (20:80 *v*/*v*), and then, vortexed for 5 min and centrifuged at 15,000 rpm for 10 min. Next, 200 μL of supernatant was transferred to a new tube and evaporated in a Vacufuge at 45 °C for 30–45 min until dry. Then, the concentrated solutes were reconstituted in 100 μL of 5% acetonitrile with 0.1% formic acid spiked with 200 ng/mL of meropenem as the internal standard [26]. Again, the samples were vortexed for 5 min and centrifuged at 15,000 rpm for 10 min. A final volume of 80 μL from the supernatant was transferred to the HPLC autosampler vial.

#### 2.5.2. Gentamicin

Deproteination of plasma samples was achieved by taking 40 μL of 100-fold-diluted plasma in LC-MS/MS water and adding 80 μL of 100% acetonitrile containing 200 ng/mL of tobramycin as the internal standard [22]. The precipitated samples were vortexed, followed by centrifugation at 15,000 rpm for 10 min. A total of 40 μL supernatant was collected and added to 40 μL of 20 mM HFBA in water, and the mixture was vortexed and transferred to autosampler vials.

For brain samples, 200 μL of 10-fold-diluted brain homogenate spiked with gentamicin was treated with 200 μL of 5% TCA in water, and then, 800 μL of 100% acetonitrile (containing 100 ng/mL of internal standard) was added. After vortexing and centrifuging the precipitated samples at 15,000 rpm for 10 min, 1 mL of supernatant was collected in a 1.5 mL centrifuge tube and completely dried in a Vacufuge at 45 °C. The dried sample was reconstituted with 100 μL of 20 mM HFBA in water and vortexed for 5 min. Then, the samples were again centrifugated at 15,000 rpm for 10 min, and 70 μL of supernatant was transferred to autosampler vials.

### 2.6. Method Validation

All the stated method validation parameters were tested following bioanalytical method validation guidance by the United States Food and Drug Administration [27].

#### 2.6.1. Selectivity

Selectivity is the extent to which the method can determine ceftazidime or gentamicin in the analyzed matrices without interference from matrix components. To ensure selectivity, blank matrix samples containing no analytes were analyzed. Additionally, the interference among the transitions of analytes and internal standards was analyzed in both neat and matrix samples.

#### 2.6.2. Linearity

The linearity of calibration curves was assessed by determining the coefficient of variation (r^2^). Linear regression analysis of the concentration–response curve using 1/x, where x is the concentration, was carried out to obtain the coefficient of variation (r^2^).

#### 2.6.3. Accuracy and Precision

Inter- and intra-day runs were performed to determine the accuracy and precision. The quality control samples were analyzed against the calibration curve for both brain and plasma matrices. Five replicates of each blank quality control, the LLOQ (lower limit of quantification), and low, medium and high concentrations of the quality controls were analyzed. Accuracy was calculated as a percentage of measured concentration over nominal concentration. Precision was calculated as a percentage of relative standard deviations (R.S.D.). The acceptable range for LLOQ was 80–120%, and for all other concentrations, it should be within the range of 85–115% as specified. The accuracy and precision were established in 3 independent runs for both the brain and plasma matrices.

#### 2.6.4. Recovery

Recovery refers to the extraction efficiency of an analytical process, reported as a percentage of the known amount of an analyte carried through the sample extraction and processing steps of the method [27]. Five replicates of the LLOQ and medium and high concentrations of the quality controls in each matrix were compared with the post-spiked samples with equivalent concentrations processed through the sample preparation method, starting with blank matrices (as a reference). The post-spiking concentrations were calculated considering the wasted analyte amounts during the sample preparation steps mentioned above. Recovery was calculated as the percentage of the ratio of the peak areas of the samples to the peak area of the reference samples.

#### 2.6.5. Freeze–Thaw Stability

For the freeze–thaw stability test, 100 ng/mL neat samples of ceftazidime and gentamicin were subjected to three freeze–thaw cycles. One cycle is defined as a minimum of 12 h of freezing at −80 °C and thawing at room temperature. The samples that underwent the freeze–thaw cycles were compared against the freshly prepared calibration curve and quality controls.

#### 2.6.6. Long-Term Storage Stability

For long-term storage stability tests, quality controls at the LLOQ and medium and high concentrations of analytes in the brain and plasma (n = 3) were stored at −80 °C for 3 months. The stored samples were then compared against freshly prepared calibration curves and quality controls of the respective matrices.

### 2.7. In Vitro Permeability Study Using iPSC-Derived Human BMECs

#### 2.7.1. iPSC Differentiation Procedure

iPSC differentiation into human BMECs was performed using an established protocol [28]. IMR90-c4-induced pluripotent stem cell line was obtained from the WiCell cell repository (WiCell, Madison, WI, USA). Undifferentiated stem cells at a density of 100,000 cells/mL were seeded on six-well tissue culture-treated plates coated with Matrigel (C-Matrigel; Corning, Corning, MA, USA) in Essential 8 medium (E8 Thermo Fisher, Waltham, MA, USA) containing 10 μM Y-27632 (Tocris, Minneapolis, MN, USA). Cells were maintained in E8 until they reached the desired cell confluency. Then, differentiation was initiated using the unconditioned medium (UM: Dulbecco’s modified Eagle’s medium/F12 with 15 mM HEPES (Thermo Fisher, Waltham, MA, USA), 20% knockout serum replacement (Thermo Fisher, Waltham, MA, USA), 1% non-essential amino acids (Thermo Fisher, Waltham, MA, USA), 0.5% Glutamax (Thermo Fisher, Waltham, MA, USA) and 0.1 mM β-mercaptoethanol (Sigma-Aldrich, St. Louis, MO, USA)) and maintained for 6 days. After 6 days, cells were incubated for two days with EC++ medium (human serum-free endothelial medium (hESFM, Thermo Fisher, Waltham, MA, USA) supplemented with 1% bovine platelet-poor plasma-derived serum (PDS, Alfa Aesar, Ward Mill, MA, USA), 10 ng/mL bFGF and 10 μM retinoic acid (Sigma-Aldrich)). After eight days of differentiation, cells were removed via Accutase (Corning) treatment and seeded as single cells on a 12-well Transwell^®^ (polyester, 0.4 μm pore size; filter area of 1.12 cm^2^, Corning) coated with a solution of collagen from human placenta (Sigma-Aldrich) and bovine plasma fibronectin (Sigma-Aldrich) (400 μg/mL collagen IV and 100 μg/mL fibronectin) at a density of 1,000,000 cells/cm^2^. After 24 h of seeding, EC—medium was added (EC medium supplemented with 1% serum derived from platelet-poor plasma). Purified brain endothelial monolayers were formed on day 10 of the experiment, and permeability barrier function tests were performed 48 h after seeding on the Transwell system. The barrier integrity of the hBMEC monolayer was ensured before performing the permeability experiment by measuring transendothelial electrical resistance (TEER) using a Millicell ERS electrode (Millipore, Bedford, MA, USA). After taking three measurements for each insert, the average TEER was obtained.

#### 2.7.2. Permeability Study on Transwell System

For the permeability experiment, 1 mg/mL of ceftazidime or gentamicin dissolved in EC—medium was added to the upper chamber of the Transwell, including the control wells without cells. The plate was incubated at 37 °C on a rocking plate. A total of 50 μL of the samples were collected from the lower chamber of the Transwell at 0-, 30-, 60-, 90- and 120-min time points, and 50 μL of fresh warmed EC—medium was replaced at each time point to maintain the same volume. The samples were prepared similarly to the plasma sample processing procedure mentioned above and analyzed in LC-MS/MS, where the plasma was replaced with a 100-fold-diluted EC—medium.

To calculate the permeability coefficient, P, the cleared volume was determined by using Equation (1):

(1) $$Cleared\;volume=\frac{C_{abluminal}\times V_{abluminal}}{C_{luminal}}$$

where *C* (abluminal) refers to the measured concentration in the abluminal compartment at a given sampling time point, *V* (abluminal) refers to the volume of the abluminal compartment, and *C* (luminal) refers to concentration in the luminal compartment. The cleared volume was calculated for the treatment groups (Transwells with hBMEC monolayer) and blank filters (without hBMEC monolayer). Linear regression analysis after plotting the cleared volume at each sampling time point over time allowed for the determination of the permeability surface area (PS) product as the slope of the regression line. Then, the permeability coefficient was obtained using Equation (2):

(2) $$P=\frac{PS}{S}$$

where *P* is the permeability coefficient, *PS* is the permeability-surface area product and *S* is the surface area of the Transwell insert in cm^2^.

Finally, the permeability of the hBMEC monolayer was corrected for the permeability of the filter insert:

(3) $$\frac{1}{P_{cells}}=\frac{1}{P_{total}}-\frac{1}{P_{filter}}$$

where *P_cells_* is the actual permeability of the monolayer, *P_total_* is the measured, uncorrected permeability of the monolayer and filter and *P_filter_* is the permeability of the empty filter.

### 2.8. In Vivo Permeability Study under Isoflurane and Ketamine/Xylazine

For the in vivo permeability study, male C57BL/6 mice (n = 5–6) were anesthetized using two different types of anesthetic agents: isoflurane and ketamine/xylazine. In the first group, 3% *v*/*v* isoflurane with 70% nitrous oxide and 30% oxygen flowed through an induction chamber at a rate of 1 L/min for the first few minutes until the animals were anesthetized. Then, 1.5–2% *v*/*v* isoflurane was maintained via a silicone face mask during the rest of the procedure. For the second group, ketamine/xylazine (100:10 mg/kg) was injected intraperitoneally. After the surgical anesthesia stage was achieved, the jugular veins were bilaterally exposed via incision of the neck for IV injections on one side and blood collection from the contralateral side. Antibiotics (ceftazidime or gentamicin) were injected intravenously at a dose of 25 mg/kg. Ceftazidime was administered as a bolus injection, while gentamicin dose was administered via short infusion (≈5 min) with a constant flow rate of 100 µL/min. A total of 50 µL of blood samples were collected at 1, 5, 10, 20 and 30 min after the drug injection. After the 30 min time point, the thorax was opened and a PE50 catheter inserted into the left ventricle of the heart for transcardiac vascular perfusion with a total volume of 20 mL at a flow rate of 2 mL/min, using phosphate-buffered saline (PBS) with heparin (10 USP Units/mL). At the beginning of the perfusion, both jugular veins were cut open to allow for the outflow of blood and perfusate. The outflow fluid was visually inspected to ensure the appearance of clear perfusion fluid towards the end of the perfusion. Animals in both groups were euthanized via decapitation, brains were removed from the skulls (without olfactory bulbs, cerebellum and brain stem), meninges were cleaned off and forebrains were weighed and homogenized in LC-MS/MS water (1:9 ratio). The homogenized brains were stored at −80 °C for the LC-MS/MS sample preparation step. Plasma was extracted through centrifugation of the blood samples at 6000 rpm for 10 min and the supernatant was collected. Plasma was diluted 100-fold in LC-MS/MS water and stored at −80 °C until sample preparation for LC-MS/MS analysis.

Values for brain uptake clearance, *K_in_*, were calculated using the following equation:

(4) $$K_{in}=C_{br}/{AUC}_{0}^{T}$$

Here, the $C_{br}$ is the brain concentration of the analytes and expressed as percentage of injected dose per gram of tissue (%ID/g). The ${AUC}_{0}^{T}$ denotes the area under the plasma concentration–time curve from time 0 to the terminal sampling time. ${AUC}_{0}^{T}$ was obtained using the logarithmic trapezoidal method after plotting the plasma drug concentrations against sampling time.

### 2.9. Statistical Analysis

GraphPad Prism 9 software (GraphPad Software, LaJolla, CA, USA) was used for graphical presentation and for statistical analysis. The data passed tests for normality in Prism, and group comparisons were performed using an unpaired *t*-test with Welch’s correction, which does not assume equal SDs. Two-way ANOVA was applied for the plasma concentration–time data analysis using the Greenhouse–Geisser correction, which does not assume sphericity. A *p*-value less than 0.05 was considered statistically significant.

## 3. Results

### 3.1. Mass Spectrometry and Chromatographic Optimization

**Figure 2:**
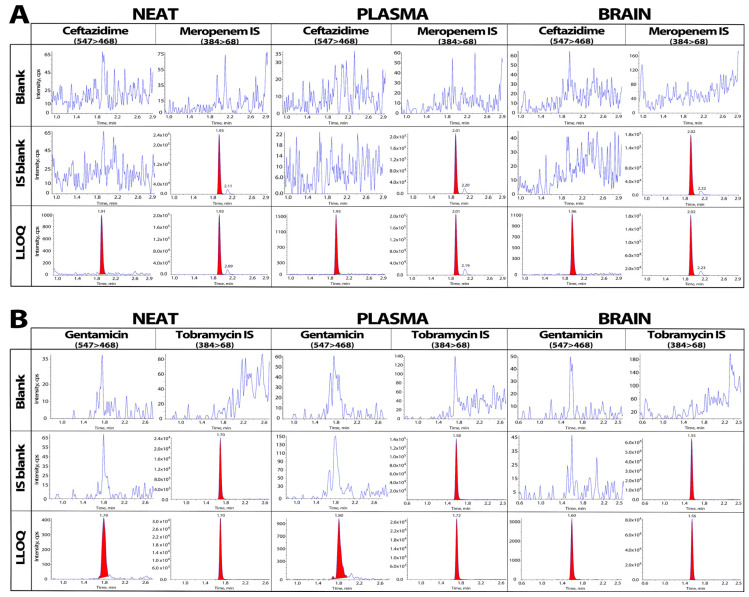
LC-MS/MS chromatograms for ceftazidime and meropenem (**A**) and gentamicin and tobramycin (**B**). Blank, blank internal standards (IS) meropenem or tobramycin, respectively, and lower limit of quantification (LLOQ) samples were prepared in neat, plasma and brain matrices. There is no interference observed between analytes and IS. For ceftazidime, the intensity of blank samples is <10% of the intensity observed in LLOQ samples (10 ng/mL for neat and plasma and 5 ng/mL for brain). For gentamicin, the intensity of blank samples is around 16–17% of the intensity observed in LLOQ samples (10 ng/mL for all matrices).

The most suitable m/z transitions for ceftazidime, gentamicin and the internal standards meropenem and tobramycin, respectively, were selected based on signal-to-noise ratio and high sensitivity (see Table 1). The peaks were stable and reproducible with multiple injections. The retention time for ceftazidime and meropenem was between 1.9 and 2.3 min, and gentamicin C2 + C2a and tobramycin retention ranged between 1.5 and 1.8 min after injection (Figure 2).

### 3.2. Selectivity

Figure 2 demonstrates that the chromatograms of the neat samples of the two analytes and the corresponding internal standards prepared in water had good selectivity with no cross-channel interference. The ceftazidime and gentamicin chromatograms at the lower limit of quantitation (LLOQ) and the blank samples containing only internal standards in the plasma and brain matrices also showed no interference transitions at the specified retention times. The baseline value for the blank samples was stable and low, at <5% and <18% of the LLOQ peak area for ceftazidime and gentamicin, respectively (Figure 2).

### 3.3. Linearity

The calibration curves were generated within the ranges of 10 to 1000 ng/mL and 5 to 400 ng/mL in the plasma and brain, respectively. We confirmed the linearity of the calibration curves with r^2^ > 0.99 for both the plasma and brain matrices across all the runs conducted in the study.

### 3.4. Accuracy and Precision

Tables S1 and S2 indicate the inter- and intra-run precision and accuracy data for ceftazidime and gentamicin in the plasma and brain samples. The accuracy and precision values were within the acceptable range specified in the Food and Drug Administration (FDA) guidelines for bioanalytical method validation [27]. The inter- and intra-day precision and accuracy (CV) for ceftazidime at four different concentrations ranged from 5.0 to 10.4% and 2.2 to 9.5% in the plasma and brain samples, respectively. Gentamicin also showed a similar range of accuracy and precision (CV) for both inter- and intra-run data, ranging from 2.3% to 9.9% for plasma samples and 2.7% to 11.22% for brain samples.

### 3.5. Recovery and Stability

The recovery rates of five replicates were between 89.9% and 102.0% over a concentration range from 10 to 1000 ng/mL in plasma samples for both ceftazidime and gentamicin. For the brain matrix, ceftazidime showed recovery rates greater than 89.9% at concentrations from 5 to 400 ng/mL, and the gentamicin recovery rates were above 81.5% at concentrations from 10 to 1000 ng/mL. Recovery was consistent across all samples, with ± SD less than 10.6% (Table S3). Figure S1 presents the results of the freeze–thaw stability study at the concentration of 100 ng/mL in neat samples. The results indicate that ceftazidime and gentamicin remained stable across three freeze–thaw cycles. The long-term storage stability test results confirmed that we could stably detect ceftazidime and gentamicin analytes after three months of storage at −80 °C with as much sensitivity as in freshly prepared samples in the brain and plasma matrices, without any significant degradation or loss (Table S4).

### 3.6. In Vitro Permeability Measurements

**Figure 3:**
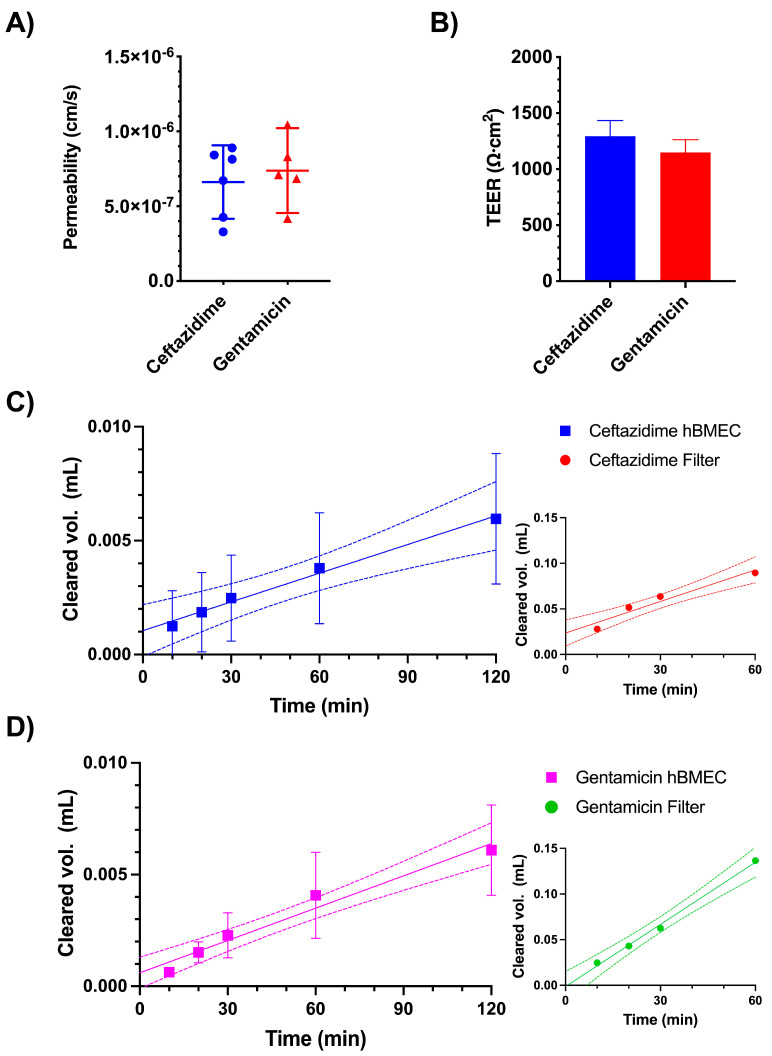
In vitro permeability analysis using iPSC-derived hBMEC cells in Transwells. The calculated permeability coefficient across the monolayer, without exposure to anesthetic agents, was measured over 2 h of incubation with the presence of ceftazidime (n = 6) and gentamicin (n = 5) in the apical chamber (**A**). Transendothelial electrical resistance (TEER) values were measured before the experiments to ensure hBMEC monolayer integrity (**B**). The permeability coefficients were calculated based on the slope of the cleared volume-versus-time graphs for the filter (without cells) and induced pluripotent stem cell (iPSC)-derived human brain microvascular endothelial cells (hBMECs) for ceftazidime (**C**) and gentamicin (**D**). Panels A and B show mean ± 95% CI; no difference was detected by the unpaired *t*-test with Welch’s correction (*p* > 0.05). Panels (**C**,**D**) show mean ± CI and linear regression lines (solid) with 95% confidence bands (dotted lines).

The in vitro permeability was evaluated using induced pluripotent stem cell (iPSC)-derived human brain microvascular endothelial cells (hBMECs) cultured using the Transwell system. The permeability coefficients (P) of ceftazidime and gentamicin were 6.62 × 10^−^^7^ ± 2.34 × 10^−^^7^ cm/s and 7.38 × 10^−^^7^ ± 2.29 × 10^−^^7^ cm/s, respectively (Figure 3A). For all the Transwells used in this experiment, we ensured consistent barrier integrity of the hBMEC monolayers by measuring the transendothelial electrical resistance (TEER) values prior to the permeability study, which were 1295 ± 139.0 Ω cm^2^ for the wells used to measure ceftazidime transport and 1149 ± 113.1 Ω cm^2^ for the wells used in the gentamicin experiment (Figure 3B). Figure 3C,D illustrate the measured clearance data across the monolayers over 120 min or blank Transwell filters over 60 min for ceftazidime and gentamicin, respectively. The *p*-values in Figure 3A correspond to the slopes of the linear regression lines after correction for the permeability of the filters without cells.

### 3.7. In Vivo Permeability: Isoflurane Exposure Compared to Ketamine/Xylazine

**Figure 4:**
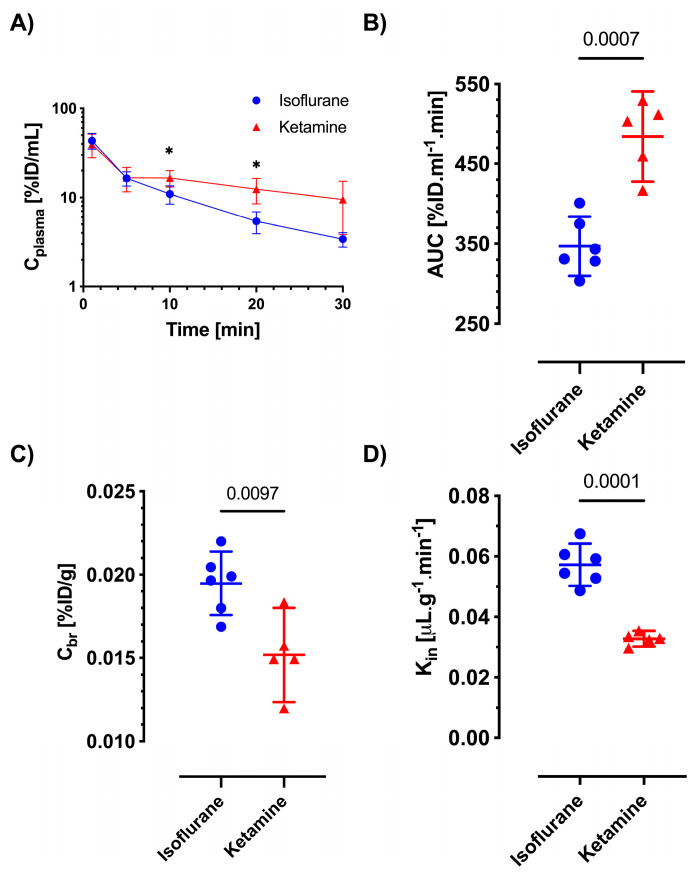
In vivo permeability for ceftazidime under isoflurane and ketamine/xylazine. Mice (n = 5–6) were anesthetized with either isoflurane (1.5–2%) or ketamine/xylazine (100:10 mg/kg) and given an IV bolus of ceftazidime (25 mg/kg). Blood was collected at different time points, and plasma–time profiles and *AUC*s after the ceftazidime injection are depicted in (**A**,**B**). Panel (**A**) represents mean values ± CI, and 2-way ANOVA was performed for statistical analysis (* = *p* < 0.05). Brain vasculatures were washed with PBS through cardiac perfusion after 30 min, and the brain concentrations were measured after euthanizing the animals (**C**). The apparent brain uptake clearance (*K_in_*) values under isoflurane and ketamine are shown in (**D**). Graphs show individual values, means and CI in (**B**–**D**), and statistical analysis was conducted using an unpaired *t*-test with Welch’s correction, not assuming equal SDs.

**Figure 5:**
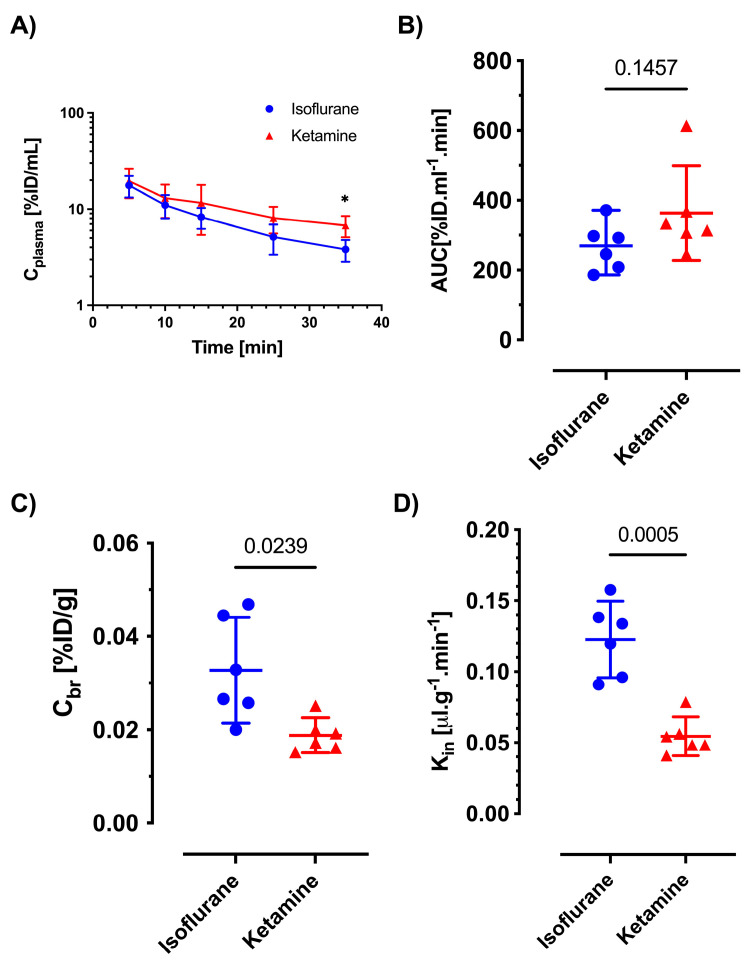
In vivo permeability for gentamicin under isoflurane and ketamine/xylazine. Mice (n = 6) were anesthetized with either isoflurane (1.5–2%) or ketamine/xylazine (100:10 mg/kg) and given an IV short infusion of gentamicin (25 mg/kg) at a rate of 100 µL/min. Blood was collected at different time points, and plasma concentration–time profiles and *AUC*s after injection are depicted in (**A**,**B**). Panel (**A**) represents mean values ± CI, and 2-way ANOVA was performed for statistical analysis (* = *p* < 0.05). Brain tissue concentrations were measured following vascular washout 30 min after gentamicin injection (**C**). The apparent brain uptake clearance (*K_in_*) values under isoflurane and ketamine are shown in (**D**). Graphs in (**B**–**D**) show individual values, means and CI. Statistical analysis was conducted using an unpaired *t*-test with Welch’s correction, not assuming equal SDs.

The plasma concentration–time profiles of both antibiotics were similar between the two anesthesia methods at earlier time points, but the mice injected with ketamine/xylazine showed higher plasma concentrations at some of the later time points, as shown in Figure 4A and Figure 5A. While there was a significant increase in ${\left.AUC\right|}_{\;0}^{30}$ for ceftazidime under isoflurane (*p* = 0.0007 vs. ketamine/xylazine; see Figure 4B), the ${\left.AUC\right|}_{\;0}^{30}$ of gentamicin was not significantly different between the isoflurane and ketamine/xylazine groups (Figure 5B). Meanwhile, in terms of brain concentration (*C_br_*), both ceftazidime and gentamicin showed significantly higher concentrations in the isoflurane group compared to the ketamine/xylazine group (*p* = 0.0097 and *p* = 0.0239, respectively; see Figure 4C and Figure 5C). The absolute concentrations in brain homogenate were 16.22 ± 1.79 ng/mL vs. 10.59 ± 1.92 ng/mL for ceftazidime and 22.35 ± 6.51 ng/mL vs. 17.75 ± 7.32 ng/mL for gentamicin (isoflurane vs. ketamine/xylazine). The brain uptake clearance (K_in_) of both ceftazidime and gentamicin increased significantly, approximately two-fold, in the isoflurane-exposed groups compared to ketamine/xylazine anesthesia, with values of 0.057 ± 0.006 μL min^−1^ g^−1^ vs. 0.033 ± 0.003 μL min^−1^ g^−1^ (*p* = 0.0001) for ceftazidime, and 0.101 ± 0.034 μL min^−1^ g^−1^ vs. 0.052 ± 0.016 μL min^−1^ g^−1^ (*p* = 0.0005) for gentamicin, respectively, as shown in Figure 4D and Figure 5D.

## 4. Discussion

To investigate the hypothesis that volatile anesthetic agents may increase the BBB permeability of potential neurotoxic drugs, ceftazidime and gentamicin were chosen, because both antibiotics have been reported to induce neurotoxicity in some patients when they accumulate in the body, e.g., due to renal impairment. Because of its hydrophilic character (log *P* = −1.6 and −3.4, respectively) [29,30], crossing of the BBB by either agent is expected to be restricted and to occur primarily through passive diffusion [31,32]. Both agents show low plasma protein binding (<15%), and there is no evidence for the involvement of uptake or efflux transporters across the BBB [31,32]. Among cephalosporins, ceftazidime has been reported to be among those with highest K_i_ values (>10 mM) for the oligopeptide transporters PEPT1 and PEPT2 [33]. The latter is expressed by the choroid plexus epithelium and by neurons, and its effect on the concentration of the cephalosporin cefadroxil in brain extracellular fluid and in brain cells has been demonstrated [34]. When studying changes in brain uptake via passive permeability, without interference from any transporters, it is therefore advantageous that ceftazidime has negligible affinity to PEPT2.

To measure the low concentrations of the antibiotics expected in brain tissue, we established highly sensitive LC-MS/MS analytical methods for these analytes. We carefully optimized the protein precipitation by using 5% TCA and/or acetonitrile to help with the extraction and recovery of gentamicin and ceftazidime from the brain matrix for analysis. Reversed-phase chromatographic separation and gradient elution achieved short total runtimes and sharp peaks. The main challenge for both antibiotics was to retain the compounds in a C18 stationary phase due to their hydrophilic nature. For ceftazidime, the problem was resolved by switching to an Accucore C18 column from an Acquity C18 BEH amide column, which has been previously used for its analysis [23]. Gentamicin, with its greater polarity, was more challenging to analyze in various biological matrices with optimum protein precipitation. In pilot studies, we found that the addition of heptafluorobutyric acid (HFBA) as an ion-pairing agent in the mobile phase [25] improved the retention and separation of gentamicin on a C18 column after trichloroacetic acid precipitation [24]. We selected stable and reproducible m/z transitions for both analytes based on multiple injections with different matrices. Meropenem and tobramycin were suitable internal standards for the analysis of ceftazidime and gentamicin, respectively, including the same retention time and no interference with the analyte transitions. The evaporation step in the sample preparation increased the sensitivity, allowing us to detect low concentrations of ceftazidime and gentamicin in the plasma and brain. The recovery data indicated no loss of analytes during all of the sample preparation steps, including the evaporation step (Table S3). Notably, we found that polypropylene vials and inserts are more suitable for gentamicin analysis, as we observed contaminations and inconsistent results using glass vials in pilot studies. The analytes and IS transition peaks provided satisfactory signal-to-noise ratios for use in the pharmacokinetic study.

All validations for the LC-MS/MS methods, including accuracy and precision, standard curves, recovery, and stability, adhered to the specifications of the bioanalytical method validation guidance currently accepted by the FDA [27].

The iPSC-derived hBMEC monolayer used here as an in vitro BBB model has been shown to exhibit excellent paracellular barrier properties, is well characterized with respect to the expression of BBB-specific transporters and carriers, and is suitable to analyze the permeability of small molecules [35,36,37], notwithstanding limitations in studies of immune mechanisms [38] and doubts regarding its endothelial characterization [39]. For the purpose of Transwell studies aimed at the analysis of the transport of substances with very low passive permeability, such as in the present experiments, it represents a viable in vitro model [28]. TEER values in BBB in vitro models of at least 1 kΩ cm^2^ are desirable for restricted paracellular permeability [40]. The values of 1295 ± 139 Ω cm^2^ and 1149 ± 113 Ω cm^2^ in the present experimental series are comparable to those in our recent study using the same iPSC-derived hBMEC model [6]. The in vitro permeability coefficients, *P*, of ceftazidime and gentamicin (6.62 × 10^−^^7^ ± 2.34 × 10^−^^7^ cm/s and 7.38 × 10^−^^7^ ± 2.29 × 10^−^^7^ cm/s, respectively) are close to the P of the [^13^C_12_]sucrose (6 × 10^−^^7^ cm/s [41]). These values are in line with the expected low permeability resulting from the physicochemical characteristics of the agents (hydrophilic structures with high numbers of hydrogen acceptors and donors, high polar surface areas and negative log *P* values; see Figure 1).

Similarly, the in vivo brain uptake studies showed values of brain uptake clearance in the range of hydrophilic BBB markers. Based on our recent data showing that ketamine/xylazine anesthesia does not affect the BBB regarding the passive permeability of hydrophilic markers in comparison to awake animals [6], we considered ketamine/xylazine anesthesia to be a suitable control condition in comparison to isoflurane for the present experiments. In ketamine/xylazine-anesthetized mice, the *K_in_* values of ceftazidime (0.033 ± 0.003 μL min^−1^ g^−1^) and gentamicin (0.052 ± 0.016 μL min^−1^ g^−1^) found in the present study are similar to the value of sucrose in our previous report (0.0612 μL min^−1^ g^−1^). Notably, ceftazidime and gentamicin showed a two-fold increase in *K_in_* under isoflurane anesthesia, resulting in a corresponding increase in brain concentrations. This is comparable to the effects seen with the passive permeability markers sucrose and mannitol [6], where these are used at tracer concentrations in plasma without relevant osmotic effect (e.g., less than 1 mM). Because neither of the antibiotics is known as a substrate of active or facilitated transport at the BBB, the presumed uptake mechanism is via passive diffusion. Therefore, we did not investigate the potential dose dependence of *K_in_*. 

Broad-spectrum cephalosporins and aminoglycosides, including ceftazidime and gentamicin, are in common clinical use, especially in intensive care units and in settings like perioperative prophylaxis [42,43,44,45]. These agents are therefore frequently administered to patients undergoing procedures under general anesthesia. Although ceftazidime is considered a relatively safe drug, incidents of neurotoxicity in patients have been documented. A recent report from the French Pharmacovigilance Database analyzed serious cases of adverse effects of different generations of cephalosporins on the CNS, and ceftazidime accounted for approximately 20% of cases [14]. Regarding gentamicin, its main neurotoxic effect manifests in the form of ototoxicity. Cochlear hair cells accumulate aminoglycosides present in endolymph fluid through mechanoelectrical transduction (MET) channels [46]. The route of uptake into endolymph is via the stria vascularis. While the capillaries of the stria vascularis express tight junctions restricting paracellular transport [47], recent evidence indicates that megalin-mediated transport at the level of the marginal epithelial cell layer is responsible for the entry of aminoglycoside into the endolymph. In contrast to ototoxicity, other CNS toxicity associated with gentamicin, e.g., encephalopathy, is less reported and its mechanistic details not well understood [19]. The results of the present study and previous work by our group and others indicate that volatile anesthetics exert acute effects on the BBB, which cause enhanced brain uptake via passive diffusion. The interaction of these agents with lipid membranes has been considered a crucial part of their pharmacological activity in neurons since the introduction of Meyer and Overton’s hypothesis at the turn of the 20th century. Altered lipid membrane structure caused by these lipophilic substances in other cell types, including the endothelial cells forming the tight barrier of the brain microvasculature, will also affect function, for instance, through higher permeability due to increased membrane fluidity [11]. These findings could be of clinical relevance and may stimulate future studies employing neurophysiological in vivo models (e.g., seizure threshold) to determine whether inhalational anesthetics enhance the risk of CNS damage, by causing an approximately two-fold increase in BBB permeability for hydrophilic small-molecule drugs with potential neurotoxicity when these are present in the blood during anesthesia. The present study was conducted in male animals only, which may be perceived as a potential limitation. However, both males and females were included in our recent publication on the effects of anesthetic agents [6], and a pilot study had not indicated sex differences. Moreover, to our knowledge, there is no evidence from the literature for sex differences in young adult rodents regarding passive BBB permeability.

In conclusion, sensitive LC-MS/MS methods for ceftazidime and gentamicin enabled accurate measurements of plasma kinetics and brain uptake. Our recent findings that volatile anesthetics change the membrane lipid fluidity and increase the BBB permeability of hydrophilic markers, including sucrose, mannitol and fluorescent dye NaFL [6], apparently also apply to drug molecules with similar physicochemical characteristics.

## Figures and Tables

**Table 1:** The optimized LC-MS/MS conditions for ceftazidime and gentamicin.

Parameters		Ceftazidime	Gentamicin
Mass/charge ratio		547.1/468.1	464.2/322.1
IS mass/charge ratio		384.1/68.0	468.3/163.2
Injection volume (μL)		5	5
Run time (min)		6	5
Flow rate (mL/min)		0.4	0.5
Retention time (min)		1.93	1.80
Column temperature (°C)		45	40
Mass spectrometer detection	Ion spray voltage	5500 V	5500 V
	Collision gas	High	High
	Curtain gas	30 psi	30 psi
	Temperature (°C)	550	500
	Ion source gas1	50 psi	50 psi
	Ion source gas2	55 psi	55 psi
	Declustering potential	80 V	100 V
	Collision energy (analyte/IS)	20 V/40 V	22 V/34 V

## Data Availability

The data presented in this study are available on reasonable request from the corresponding author.

## Supplementary Materials

The following supporting information can be downloaded at: https://www.mdpi.com/article/10.3390/pharmaceutics16010135/s1, Figure S1: Freeze–thaw stability results for ceftazidime (A) and gentamicin (B); Table S1: Accuracy and precision for plasma samples; Table S2: Accuracy and precision for brain samples; Table S3: Recovery for brain and plasma; Table S4: Long-term stability test in plasma and brain.
