# Supplementary material for: Higher Brain Uptake of Gentamicin and Ceftazidime under Isoflurane Anesthesia Compared to Ketamine/Xylazine
Source: Pharmaceutics. 2024 Jan 19;16(1):135. doi: 10.3390/pharmaceutics16010135 (PMC10820362; doi:10.3390/pharmaceutics16010135)
Supplement: Supplementary file 1 [file pharmaceutics-16-00135-s001.zip › pharmaceutics-2802176-supplementary.pdf]

Supplementary Materials

Table S1. Accuracy and precision for plasma samples

Analytes	Spiked concentration (ng/mL)	Intra-day				Inter-day			
		Measured concentration (ng/mL)	STD DV	Accuracy (%)	Precision (RSD, %)	Measured concentration (ng/mL)	STD DV	Accuracy (%)	Precision (RSD, %)
Ceftazidime	10	10.37	1.08	103.70	10.4	10.57	1.00	105.70	9.6
	20	20.13	1.00	100.65	5.0	19.58	1.46	97.90	7.4
	100	100.38	8.68	100.38	8.7	96.85	7.74	96.85	8.0
	1000	1032.54	104.90	103.25	10.2	1007.69	87.02	100.77	8.6
Gentamicin	20	21.88	1.76	109.41	8.04	21.89	2.46	109.46	11.22
	100	103.01	2.82	103.01	2.74	99.60	6.34	99.60	6.37
	500	528.14	26.34	105.63	4.99	519.27	36.51	103.85	7.03
	800	897.55	30.91	112.19	3.44	823.88	81.00	103.02	9.83

Table S2. Accuracy and precision for brain samples

Analytes	Spiked concentration (ng/mL)	Intra-day				Inter-day			
		Measured concentration (ng/mL)	STD DV	Accuracy (%)	Precision (RSD, %)	Measured concentration (ng/mL)	STD DV	Accuracy (%)	Precision (RSD, %)
Ceftazidime	10	10.37	1.08	103.7	10.4	10.57	1	105.7	9.6
	20	20.13	1	100.65	5	19.58	1.46	97.9	7.4
	100	100.38	8.68	100.38	8.7	96.85	7.74	96.85	8
	1000	1032.54	104.9	103.25	10.2	1007.69	87.02	100.77	8.6
Gentamicin	20	21.88	1.76	109.41	8.04	21.89	2.46	109.46	11.22
	100	103.01	2.82	103.01	2.74	99.6	6.34	99.6	6.37
	500	528.14	26.34	105.63	4.99	519.27	36.51	103.85	7.03
	800	897.55	30.91	112.19	3.44	823.88	81	103.02	9.83

Table S3. Recovery for brain and plasma

Ceftazidime				Gentamicin		
Spiked concentration (ng/mL)	Plasma Recovery (%, mean ±SD)	Spiked concentration (ng/mL)	Brain Recovery (%, mean ±SD)	Spiked concentration (ng/mL)	Plasma Recovery (%, mean ±SD)	Brain Recovery (%, mean ±SD)
10	96.2 ±6.2	5	89.9 ±10.6	10	92.7 ±10.2	81.5 ±0.6
100	102.0 ±4.8	50	94.4 ±9.8	100	102.7 ±0.3	90.2 ±3.9
1000	93.7 ±7.3	400	91.0 ±10.3	1000	88.5 ±3.2	100.8 ±2.4

Table S4. Long-term stability test in plasma and brain

Analytes	Spiked concentration (ng/mL)	Long-term stability in plasma			Long-term stability in brain		
		Measured Concentration (ng/mL)	Accuracy (%)	Precision (RSD, %)	Measured Concentration (ng/mL)	Accuracy (%)	Precision (RSD, %)
Ceftazidime	10	9.8	98.4	3.9	10.6	106.3	9.9
	100	92.3	92.3	8	88	88	2.3
	1000	925.1	92.5	6.9	956.5	95.7	12
Gentamicin	10	9.1	91	13.5	9.8	98	2.1
	100	101.3	101.3	0.5	105.8	105.8	1.5
	1000	1101.8	110.2	2.9	1030.8	103.1	3.7

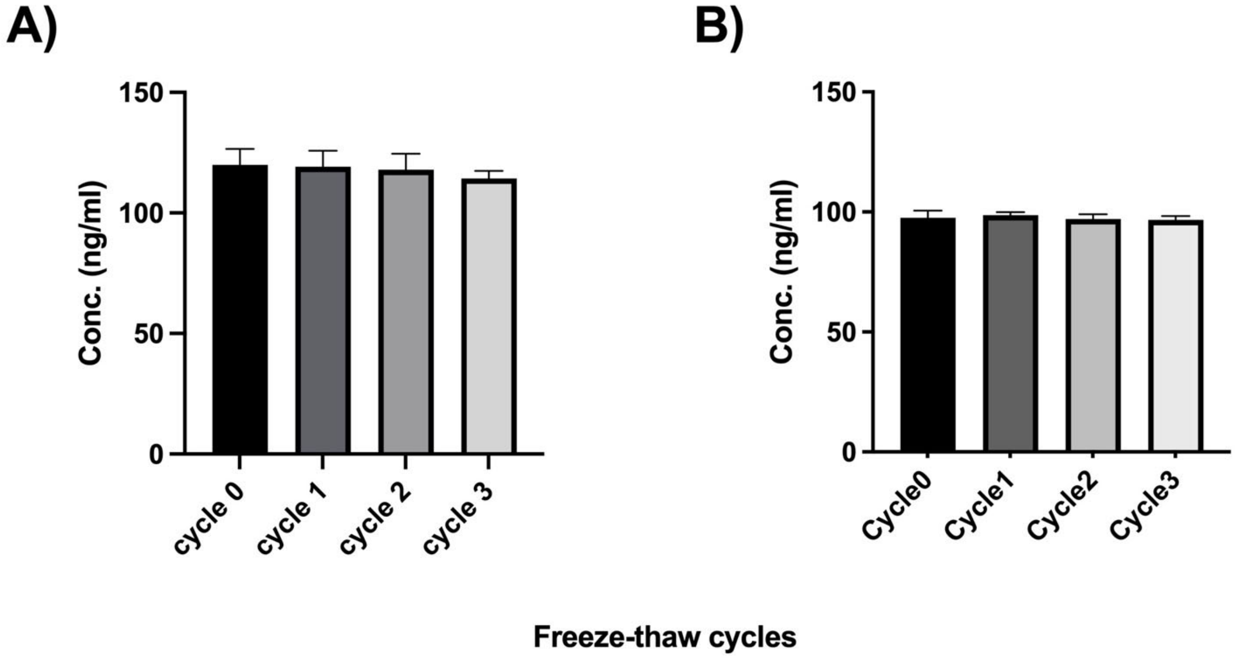

Figure S1. Freeze-thaw stability results for ceftazidime (A) and gentamicin (B). Samples were prepared at a concentration of 100ng/mL in neat and underwent freeze-thaw cycles up to three times. Cycle 0 and calibration samples were freshly prepared on the same day of analysis. Three replicates of samples from each cycle were evaluated for statistical analysis. The sensitivity for detecting both drugs was not significantly decreased after three times of the freeze-thaw process. Graphs show means ± 95% CI. Statistical analysis by one-way ANOVA, $P > 0.05$, n = 3.
